# Decreased cervical epithelial sensitivity to nonoxynol-9 (N-9) after four daily applications in a murine model of topical vaginal microbicide safety

**DOI:** 10.1186/2050-6511-13-9

**Published:** 2012-10-01

**Authors:** Karissa Lozenski, Robert Ownbey, Brian Wigdahl, Tina Kish-Catalone, Fred C Krebs

**Affiliations:** 1 Department of Microbiology and Immunology, and Center for Molecular Therapeutics and Resistance, Center for Sexually Transmitted Disease, Institute for Molecular Medicine and Infectious Disease, Drexel University College of Medicine, 245 N. 15th Street, Philadelphia, PA, 19102, USA; 2 Department of Pathology & Laboratory Medicine, Drexel University College of Medicine, 245 N. 15th Street, Philadelphia, PA, 19102, USA

**Keywords:** Microbicide, N-9, Cervix, Mouse, Toxicity

## Abstract

**Background:**

The disappointing clinical failures of five topical vaginal microbicides have provided new insights into factors that impact microbicide safety and efficacy. Specifically, the greater risk for human immunodeficiency virus type 1 (HIV-1) acquisition associated with multiple uses of a nonoxynol-9 (N-9)-containing product has highlighted the importance of application frequency as a variable during pre-clinical microbicide development, particularly in animal model studies.

**Methods:**

To evaluate an association between application frequency and N-9 toxicity, experiments were performed using a mouse model of cervicovaginal microbicide safety. In this model system, changes in cervical and vaginal epithelial integrity, cytokine release, and immune cell infiltration were assessed after single and multiple exposures to N-9.

**Results:**

After the initial application of N-9 (aqueous, 1%), considerable damage to the cervical epithelium (but not the vaginal epithelium) was observed as early as 10 min post-exposure and up to 8 h post-exposure. Subsequent daily exposures (up to 4 days) were characterized by diminished cervical toxicity relative to single exposures of like duration. Levels of pro-inflammatory cytokines released into the cervicovaginal lumen and the degree of CD14-positive immune cell infiltration proximal to the cervical epithelium were also dependent on the number of N-9 exposures.

**Conclusions:**

Rather than causing cumulative cervical epithelial damage, repeated applications of N-9 were characterized by decreased sensitivity to N-9-associated toxicity and lower levels of immune cell recruitment. These results provide new insights into the failure of N-9-based microbicides and illustrate the importance of considering multiple exposure protocols in pre-clinical microbicide development strategies.

## Background

The global human immunodeficiency virus type 1 (HIV-1) epidemic currently includes approximately 33 million HIV-1-infected people worldwide, with a particularly high incidence of infection (~23 million individuals) in Sub-Saharan Africa [1]. Since the discovery of HIV-1 over 30 years ago, the face of this global epidemic has changed dramatically, with heterosexual intercourse now considered the predominant route for the spread of the virus [1]. As a result, women are at much greater risk for acquiring HIV-1 and have a much greater need for methods that effectively reduce or eliminate the risk of infection during sexual intercourse. Although condoms (male and female) are highly effective barrier methods, they are not female-controlled and are unlikely to be used with great adherence in developing countries. To answer the critical need for effective female-controlled methods of protection, continued efforts are being directed toward the development of microbicides. A microbicide is a chemical entity that can be applied vaginally or rectally to eliminate or reduce the risk of HIV-1 transmission. Efforts to develop topical vaginal microbicides have resulted in the advancement of many candidate microbicide compounds through pre-clinical studies and clinical trials of both safety and efficacy [2-4].

Unfortunately, clinical trials involving the microbicides COL-1492 (nonoxynol-9 or N-9), Savvy (C31G), Ushercell (cellulose sulfate), Carraguard (carrageenan), and PRO 2000 [5-7] failed to demonstrate any product efficacy despite promising activities in pre-clinical studies and apparently acceptable levels of safety in early clinical studies. The results of these failed trials emphasized the urgent need for more stringent pre-clinical protocols, with emphasis on microbicide safety and the use of non-human primate models to evaluate the efficacy and safety of potential microbicides [8].

The clear need for more thorough pre-clinical evaluations is particularly apparent in retrospective analyses of the development of N-9 as a microbicide [9]. Pre-clinical assessments of N-9 failed to predict the inability of N-9 to inhibit HIV-1 transmission and the adverse effects of N-9 exposure on the risk of infection*.* Early in vitro studies of N-9, which has been widely used as a spermicidal agent for more than 40 years [9], yielded promising results, demonstrating that N-9 possessed broad-spectrum activity against several sexually transmitted disease (STD) pathogens, including *Chlamydia trachomatis*, *Neisseria gonorrhoeae*, herpes simplex virus type 2 (HSV-2), and HIV-1 [10-18]. The widespread and apparently safe use of N-9 as a human contraceptive agent further supported the development of this compound as a topical microbicide. As a consequence, N-9 was advanced into human clinical trials. However, the final phase 2/3 clinical trial of N-9 (formulated as COL-1492 with 52.5 mg N-9 per treatment dose) demonstrated that high frequency use of this product was associated with an almost 2-fold greater risk of HIV-1 acquisition [5]. Increased HIV-1 infection after N-9 application, in hindsight, has been attributed to the disruption of the cervicovaginal epithelial barrier as well as inflammation and irritation associated with N-9 application [9,19-22]. While these findings ended the further development of N-9 as a microbicide, they also raised new questions about mechanisms by which potential topical microbicides can fail.

The apparent association between N-9 application frequency and increased risk of HIV-1 infection prompted an expansion of our previous N-9 toxicity studies involving a Swiss Webster mouse model of cervicovaginal toxicity. The value of this model system was demonstrated in investigations that reiterated the clinical toxicity of N-9 (after a single application) and paralleled indications of irritation associated with topical application of 1.7% C31G [19,23,24]. Furthermore, mouse model experiments involving topical vaginal application of N-9 clearly demonstrated that (i) N-9-associated tissue damage was greatest approximately two to four hours post-application, (ii) epithelial damage was limited to the cervix, and (iii) repair and regeneration of cervical epithelial tissues was essentially complete 24 h after a single application of N-9 [19].

The present studies were conducted using the mouse model of microbicide toxicity to examine the effects of multiple daily exposures to N-9. These experiments were designed to provide a comprehensive assessment of changes in cervicovaginal integrity with respect to post-application exposure duration and the number of topical applications of N-9. The results of these investigations indicated that (i) multiple exposures to N-9 resulted in diminished cervical sensitivity to N-9 application relative to the initial exposure and (ii) cytokine release and CD14-positive cell infiltration subsequent to N-9 exposure varied with the number of exposures. These studies provide new insights into the mechanisms underlying the failure of N-9 as a microbicide and suggest a new parameter for assessing future microbicide candidate molecules.

## Methods

### Animals

Five- to six-week-old female outbred Swiss-Webster mice (CFW®) were utilized for all experiments (Charles River Laboratories International, Inc., Wilmington, MA). Mice were synchronized 7 and 3 days prior to the start of each experiment with a 0.2 mL subcutaneous injection of Depo-Provera® (Pharmacia and Upjohn Company) diluted in Lactated Ringer’s Solution (Baxter) for a final dose of 3 mg/animal. Prior to intravaginal application, mice were anesthetized with a formulation of ketamine/xylazine (100–200 mg/kg and 5–10 ng/kg, respectively). Anesthetized animals then received a single intravaginal inoculation (60 μL) of saline or N-9 diluted in saline. Mice treated with saline alone were included as controls at all time points to evaluate the normal tissue morphology in the cervicovaginal mucosa. After treatment, mice were humanely sacrificed and the cervicovaginal tracts were surgically excised and prepared for histological examination. Each experiment evaluated three animals at each time point within each test group. All animal studies conformed to the “Guiding Principle in the Care and Use of Animals” approved by the American Physiological Society, and were approved by The Drexel University College of Medicine Institutional Animal Care and Use Committee (IACUC).

### Multiple exposure experiments

**Figure 1:**
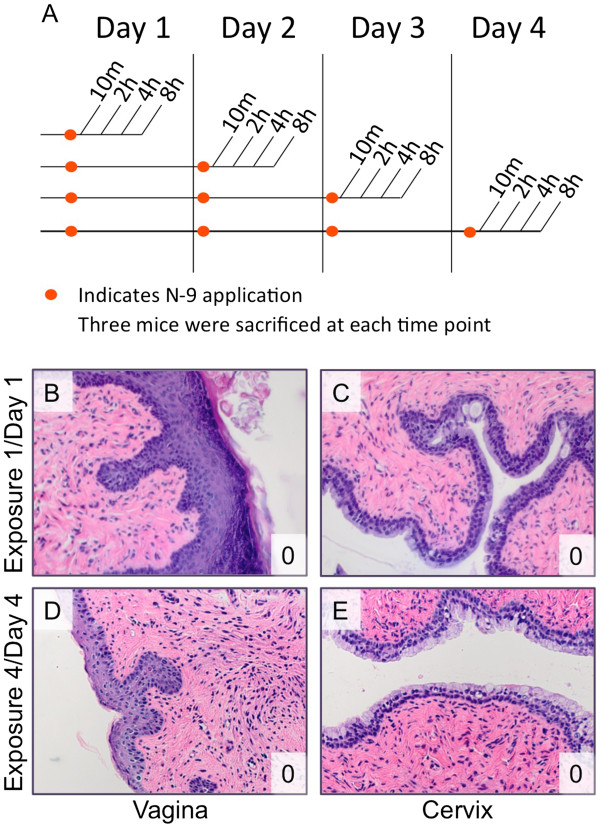
**(A) Multiple exposure experimental timeline and cervicovaginal tissue exposure controls.** Female Swiss Webster mice were exposed to N-9 daily for four days, resulting in four experimental groups of mice. Within each exposure group, subsets of mice were sacrificed at 10 min, 2 h, 4 h, or 8 h after exposure. (**B**-**E**) One mouse was exposed to saline and sacrificed after each post-exposure period. Photo micrographs (40× magnification) of representative tissues from the 2 h time point are shown. Vaginal (**B**, **D**) and cervical (**C**, **E**) epithelial tissues were examined for damage after a single exposure (**B**, **C**) or after 4 exposures (**D**, **E**) to saline. The tissue damage score (see also Table 1) is shown in the lower right corner of each panel.

The previously described single application protocol [23,25] was adapted to investigate the effects of multiple N-9 exposures. In the first set of experiments, four consecutive daily applications of N-9 were administered to the mice. This protocol parallels the FDA standardized rabbit vaginal irritation (RVI) study design, which utilizes 10 consecutive daily applications. Unlike the RVI protocol, however, the present experimental design also included intermediate assessments of tissue damage at acute post-application time points. Specifically, animals were sacrificed at acute exposure durations of 10 min, 2 h, and 4 h, and at longer exposure times of 8 h and 24 h following each application for additional and more stringent assessments of cervicovaginal toxicity (Figure 1A). These acute exposure durations were previously used to characterize the onset and development of epithelial damage following single microbicide application [23,25]. In addition, these exposure durations are presumed to be within a likely window of STD pathogen exposure and infection. The longer exposure durations have been shown to be important for characterizing the time course of epithelial repair and subsequent tissue inflammation.

Prior to excision of the reproductive tracts, animals were vaginally lavaged using 150 μl Lactated Ringer’s Solution (Baxter) by reverse pipetting. Lavage fluids were collected and stored at −80°C prior to analyses of cytokine content. The cervicovaginal tract was then excised, formalin-fixed, and embedded in paraffin using standard procedures. Tissue sections set aside for further processing were chosen to show epithelial tissues from the lower vaginal tract and the cervix. Sections were stained with hematoxylin and eosin (H&E) for assessment of tissue damage; representative fields from each treatment group were imaged and documented using deconvolution microscopy.

### Measurement of cytokine levels in vaginal lavage fluids

Cytokine levels in collected vaginal lavages were measured using a Luminex 100/200 instrument (Luminex Corporation, Austin, TX). Two murine pro-inflammatory cytokines – interleukin-1β (IL-1β) and interleukin-6 (IL-6) – were measured using commercially available plates (Millipore). Lavage samples were thawed on ice and then centrifuged for 5 minutes at a high speed to pellet any debris or mucus and to prevent plate filter clogging. Samples (25 μL per well) were added to the plate in duplicate and processed as described by the manufacturer. Results were analyzed using Xponent 3.1 software (Luminex) and graphed using Microsoft Excel to visualize changes in cytokine levels.

### Immunohistochemical staining for immune cell localization

Paraffin-embedded tissue sections were deparaffinized and rehydrated using xylene, an ethanol gradient, and deionized water as per standard protocol. Antigen retrieval was performed using trypsin in a humidified chamber followed by steaming while treating tissue with target retrieval solution (Dako S1700) for 30 min. Following this incubation, the tissue sections were treated with hydrogen peroxide and then blocked with R.T.U. normal horse serum (Vector). Primary anti-CD14 antibody (Abcam) was applied at a 1:500 dilution followed by the secondary antibody (ImmPRESS Reagent Kit peroxidase, Vector MP-7401). After the incubation with the secondary antibody, visualization of the cells was performed using the DAB:Peroxidase Substrate Kit (Vector SK-4100). The tissue was then counterstained using hematoxylin (Vector H3401) and was processed through an alcohol gradient and xylene before application of a coverslip mounted using cytoseal XYL mounting media (Richard Allan Scientific 8312–4). Staining was performed by Paragon Bioservices. Levels of staining were assessed qualitatively in sections prepared from three mice at each time point.

## Results

### Toxicity after a single N-9 exposure is localized to the cervix

The importance of assessing the impact of multiple exposures of N-9 on epithelial integrity and inflammation was illustrated by the negative consequences of frequent N-9 exposures reported in clinical trials [20,26]. To determine if multiple applications of N-9 affected cervicovaginal tissue sensitivity and subsequent recovery rates of the damaged epithelium, Swiss Webster mice were treated with 1% unformulated N-9 once daily for 4 consecutive days. Following each application, a subset of mice was sacrificed and cervicovaginal tissues were harvested 10 min, 2 h, 4 h, and 8 h after each application and assessed for morphological damage (Figure 1A). The tissues were scored visually according to a four-point tissue scoring system (Table 1).

**Table 1:** Scoring system used for the assessment of cervicovaginal epithelial tissue damage subsequent to N-9 exposure

**Score**	**Description of epithelial damage**
0	No epithelial disturbances or sloughing of epithelial cells
1	Light epithelial damage and disruption – localized loss of tissue integrity and epithelial sloughing over less than 5% of the epithelial surface, which is otherwise contiguous and intact
2	Moderate epithelial damage and disruption – Multiple areas of epithelial disturbance representing 5-25% of the total epithelial surface and small regions of sloughing that expose the basal cell layer
3	Severe epithelial damage and disruption – Sloughing over large sections of the epithelial surface (> 25%) that exposes the basal cell layer

In control animals, saline application caused no damage to the cervicovaginal epithelium. At 2 h post-exposure, the vaginal (Figure 1B) and cervical (Figure 1C) epithelia were unaffected by a single exposure to saline. Similarly, no damage to the vaginal (Figure 1D) or cervical (Figure 1E) epithelia was apparent after four daily exposures to saline. Similar results were obtained at days 2 and 3, and after post-exposure durations shorter (10 min) or longer (4 h and 8 h) than 2 h (data not shown). Saline controls were considered representative of healthy tissue, since mock-exposed tissues and saline-exposed tissues were indistinguishable (data not shown).

**Figure 2:**
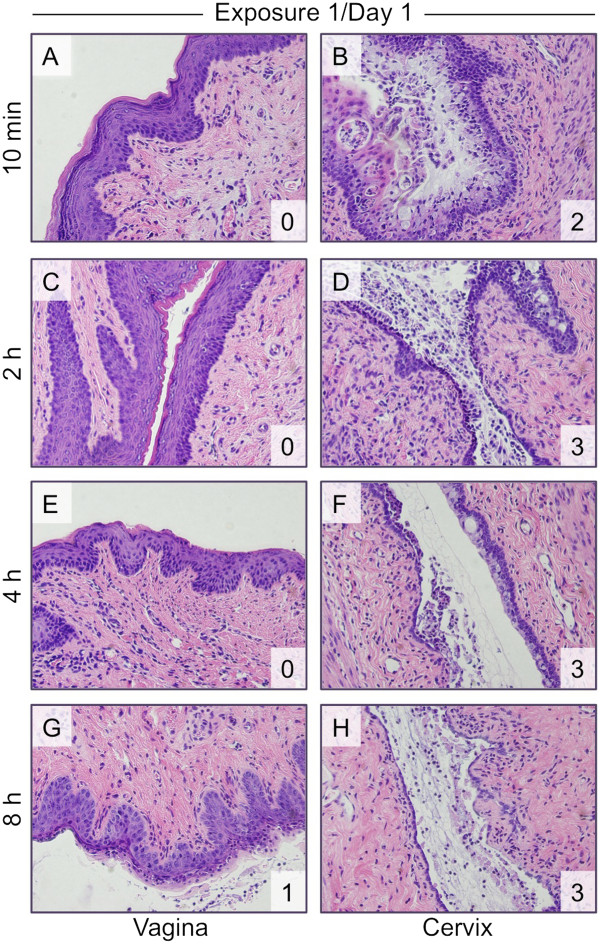
**After a single exposure to N-9, severe toxicity was observed in the cervix.** Mice were exposed once to unformulated N-9 at a concentration of 1% for the indicated durations. H&E-stained tissue sections (shown at 40× magnification) were assessed for vaginal (**A**, **C**, **E**, **G**) and cervical (**B**, **D**, **F**, **H**) epithelial toxicity and scored. The tissue damage score (see also Table 1) is shown in the lower right corner of each panel.

A single exposure to N-9 also had a minimal effect on vaginal epithelial integrity. There was little to no damage to the vaginal epithelium detectable 10 min after a single exposure to 1% N-9 (Figure 2A), despite the concurrent appearance of moderate to severe damage in the cervix (Figure 2B). Although some indications of toxicity were apparent in the upper layers of the vaginal epithelium at 2 h post-exposure, this damage was very limited; the majority of the lower vaginal epithelium appeared to be intact (Figure 2C). At 4 h post-application, the vaginal epithelium appeared to be unaffected by N-9 exposure (Figure 2E). At 8 h following the single application of N-9, light tissue sloughing was observed in the vagina (Figure 2G).

In sharp contrast, damage to the cervix was readily apparent by 10 min after a single application of N-9 (Figure 2B), with swelling of cells in the columnar epithelium, sloughing of the superficial layers of the epithelium, and presumed apoptosis. Severe sloughing across most of the superficial epithelium (leaving the basal layer intact) was consistently observed in the cervix 2 h post-application (Figure 2D). By 4 h post-exposure, there was scant superficial epithelium remaining with some foci showing complete epithelial denudation (Figure 2F). At 8 h post-exposure, very little of the superficial columnar cell layer of the cervical epithelium remained, leaving large areas of the deeper basal cell layer almost completely exposed. The majority of the mucosa contained only one or two layers of basal cells with larger areas of complete epithelial denudation (Figure 2H). By 24 h after N-9 application, however, the cervical epithelium more closely resembled saline-exposed tissue, suggesting a process of tissue repair during the preceding 16 hours (data not shown).

### Cervical sensitivity to N-9 exposure decreases with increasing exposure number

**Figure 3:**
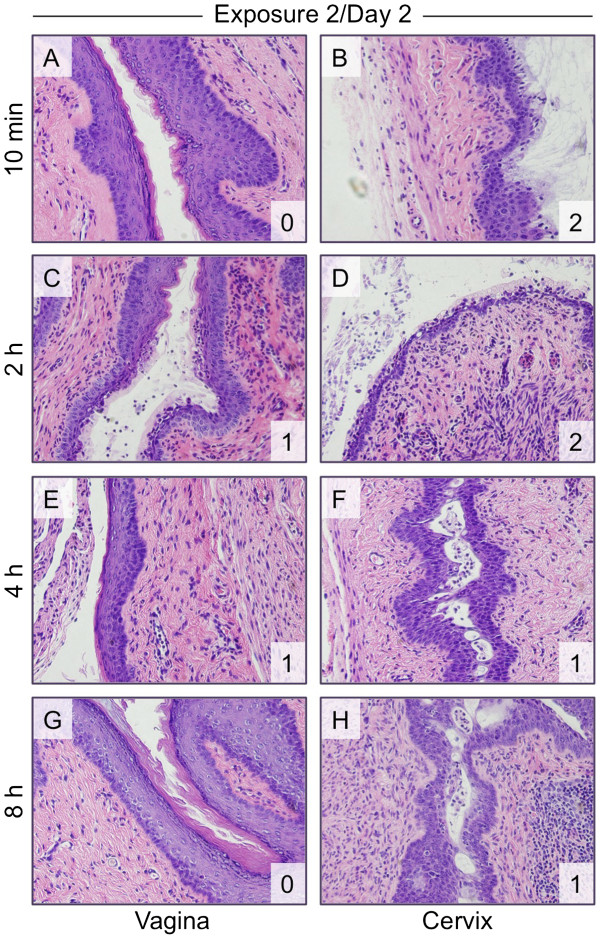
**Cervical epithelial damage is still apparent after two daily exposures to N-9.** Mice were exposed twice to unformulated 1% N-9 and sacrificed after the indicated post-exposure intervals following the second exposure. H&E-stained tissue sections (shown at 40x magnification) were assessed for vaginal (**A**, **C**, **E**, **G**) and cervical (**B**, **D**, **F**, **H**) epithelial toxicity and scored. The tissue damage score (see also Table 1) is shown in the lower right corner of each panel.

Assessments of tissue damage after the second exposure to N-9 continued to reveal minimal damage to the vaginal tissue and considerable toxicity in the cervix (Figure 3). Damage to the vagina after the second exposure was limited and isolated to the upper layers of the vaginal epithelium at 2 h post-exposure (Figure 3C). At 4 h after the second application, however, the majority of the vaginal epithelium appeared to have a more compact histological presentation (Figure 3E). Although the integrity of the continuous epithelial barrier remained intact, the cell morphology appeared denser and more tightly connected. Damage to the cervix at 10 min and 2 h post-exposure was moderate, but less severe than after the first exposure (Figure 3B and D). However, moderate shedding of the epithelial layer was evident and occasional breaks in the epithelial lining, which exposed the lamina propria, were observed. Cervical epithelial repair was again evident by 24 h post-application (data not shown).

**Figure 4:**
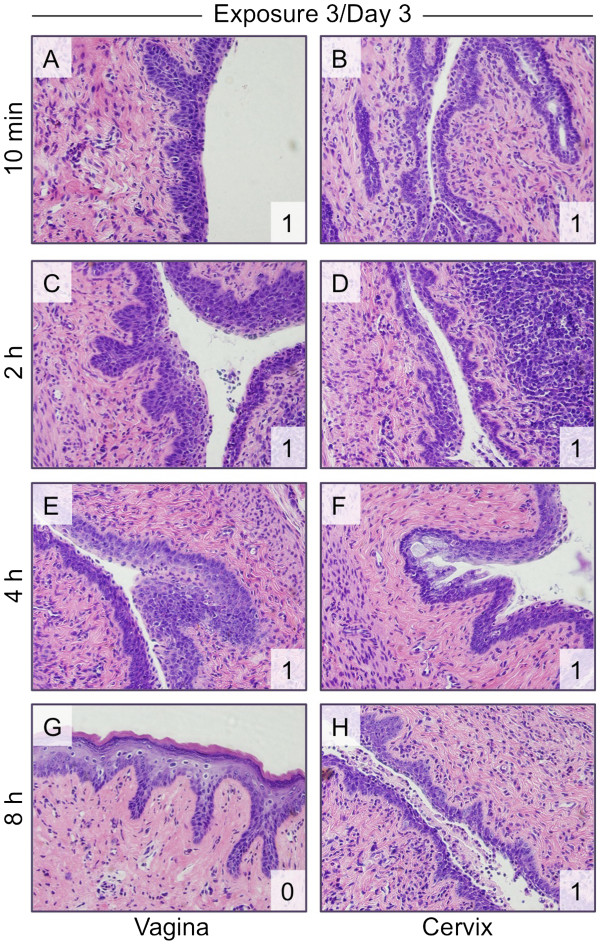
**Cervical epithelial damage is still apparent after three daily exposures to N-9.** Mice were exposed once daily over three days to unformulated 1% N-9 and sacrificed after the indicated post-exposure intervals following the third exposure. H&E-stained tissue sections (shown at 40× magnification) were assessed for vaginal (**A**, **C**, **E**, **G**) and cervical (**B**, **D**, **F**, **H**) epithelial toxicity and scored. The tissue damage score (see also Table 1) is shown in the lower right corner of each panel.

After the third N-9 exposure (Figure 4), the vaginal epithelium again appeared more compact and thinner (relative to saline-exposed tissue), especially on the lower layers of the stratified squamous epithelium. The cellular structure of the upper stratified layers appeared to have a looser configuration, indicating that cells may have been in the process of being shed. However, the vaginal tissues were still continuous and intact, with no breaks in the tissue. Damage to the cervix after the third exposure was less at 10 min (Figure 4B) and 2 h post-exposure (Figure 4D) relative to the damage observed after like durations on day 2.

**Figure 5:**
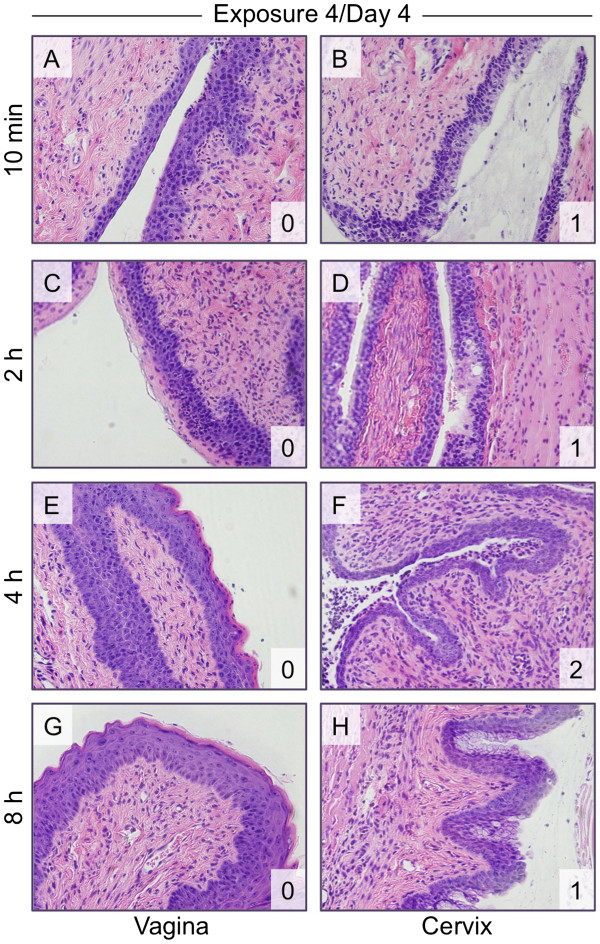
**Cervical epithelial damage after four exposures to N-9 is considerably less relative to the damage caused by a single exposure.** Mice were exposed once daily for four days to unformulated 1% N-9 and sacrificed after the indicated post-exposure intervals following the fourth exposure. H&E-stained tissue sections (shown at 40× magnification) were assessed for vaginal (**A**, **C**, **E**, **G**) and cervical (**B**, **D**, **F**, **H**) epithelial toxicity and scored. The tissue damage score (see also Table 1) is shown in the lower right corner of each panel.

Following the fourth exposure, N-9 toxicity was evident again predominantly in the cervix (Figure 5). The vaginal epithelium was generally intact, with minimal cellular shedding in the upper layers of the mucosa at 10 min and 2 h post-application (Figure 5A and C). The lower layers of stratified squamous epithelium still exhibited a more compact appearance. However, tissues analyzed at 4 h and 8 h post-exposure were more similar to the control tissues (Figure 5E and G). As was observed on day 3, the cervical epithelium appeared to be more tolerant to N-9 exposure after the fourth application relative to cervical epithelial tissues exposed once (day 1) or twice (day 2). With each daily exposure, the morphology of the cervix changed to a more compact structure, and appeared as a multi-layered structure at all time points after the fourth N-9 application (Figure 5B, D, F, and H). After the fourth application of 1% N-9, mucous-producing cells were absent in more than 50% of the N-9-exposed mice despite the recovery of the endocervical epithelium and reconstruction of an intact epithelial barrier over the lamina propria. This observation suggests the absence of normal mucous secretion. When tissue sections from the cervix were scored with respect to the degree of epithelial damage (Table 1), cervical epithelial damage scores at all four time points after the fourth daily exposure were significantly lower than corresponding damage scores recorded after the initial exposure on Day 1 (Table 2).

**Table 2:** Average damage scores for cervical epithelial tissues exposed to N-9

	**N-9 exposure duration**			
	**10 min**	**2 h**	**4 h**	**8 h**
**Exposure 1/Day 1**	1.3 ± 0.3	3.0 ± 0.0	3.0 ± 0.0	3.0 ± 0.0
**Exposure 4/Day 4**	0.0 ± 0.0	1.0 ± 0.0	1.7 ± 0.3	0.7 ± 0.3
**P value, Day 1 vs. Day 4**	0.016	<0.0001	0.016	0.002

After the first or the fourth daily exposure to N-9, mice were sacrificed following the indicated post-exposure intervals. Scores (as described in Table 1) were assigned to cervical tissue sections after multiple fields from three replicate mice at each time point within each exposure group were examined microscopically. Each value is expressed as an average score ± standard error. Significant differences between scores on Days 1 and 4 are indicated by p values less than 0.05 (as calculated by an unpaired Student’s *t*-test).

### Pro-inflammatory cytokine release varies after multiple N-9 exposures

Cervicovaginal lavages were also collected during the multiple exposure experiments to assess the release of cytokines subsequent to single or multiple exposures to N-9. We hypothesized that comparisons of pro-inflammatory cytokines released on days 1 and 4 would reveal notable differences, given the considerable and significant histological differences between cervical tissues subjected to one or four daily exposures to N-9. Because previous in vitro, rabbit, and human studies identified IL-1β and IL-6 as significant predictors of cervicovaginal toxicity following single or multiple exposures to N-9 [27,28], the present studies were focused on these factors.

**Figure 6:**
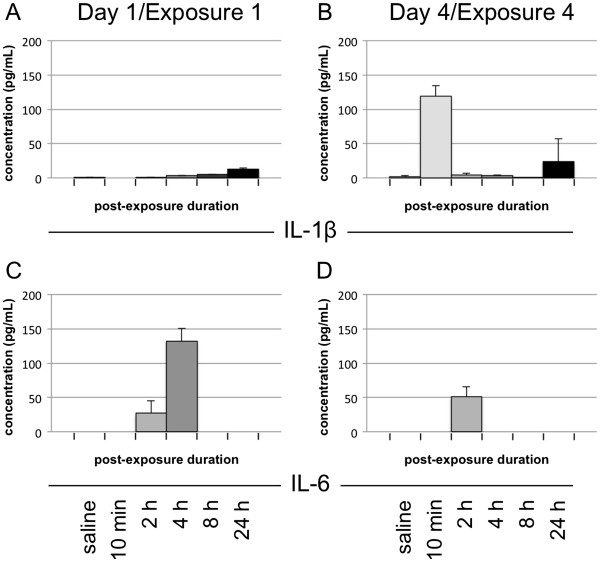
**IL-1β and IL-6 are differentially released following single and multiple exposures to N-9.** Female Swiss Webster mice were exposed once or once daily over 4 days to unformulated 1% N-9 and sacrificed after the indicated post-exposure intervals following the last exposure. Prior to sacrifice, saline cervicovaginal lavages were collected from the mice and analyzed for the presence of IL-1β (**A**, **B**) or IL-6 (**C**, **D**) using Luminex technology. The absence of a bar indicates no detectable cytokine release over background levels. Error bars indicate standard deviations on replicate data points.

Analyses of IL-1β release demonstrated that the first exposure to N-9 caused a small but steady increase in IL-1β protein release into the cervicovaginal lumen, with concentrations peaking at approximately ~10 pg/ml at 24 h post-exposure (Figure 6A). In contrast, IL-1β concentrations after the fourth N-9 exposure (Figure 6B) rapidly increased from baseline to ~115 pg/ml at 10 min post-exposure but returned to pre-exposure levels by 2 h. A second but smaller release (~20 pg/ml) was detected at 24 h post-exposure. Interestingly, the levels of IL-1β release did not appear to correspond with the severity of cervical epithelial damage. Despite the appearance of moderate to severe damage between 10 min and 8 h post-exposure on day 1, increases in IL-1β were minimal. Conversely, the minimal cervical epithelial damage following the fourth exposure was accompanied by a large but transient increase in IL-1β release.

In contrast, IL-6 levels were consistent with the degree of cervical epithelial damage. On day 1 (Figure 6C), IL-6 release was first detected at 2 h post-exposure and peaked at 4 h (~130 pg/ml) before returning to baseline levels. After four daily N-9 applications (Figure 6D), IL-6 release was detected only at 2 h post-exposure at a considerably lower concentration (~50 pg/ml) relative to day 1. Both IL-6 release patterns paralleled the time course and severity of N-9-associated cervical epithelial damage. On day 1, the peak of IL-6 release corresponded with the severe damage to the cervical epithelia noted at 2–4 h post-exposure. On day 4, the comparatively lower level of IL-6 release was consistent with the reduced severity of epithelial damage and apparent tolerance to N-9 exposure, and coincided with the modest increase in epithelial damage observed at 4 h post-exposure.

### CD14+ cell infiltration in the cervix increases following a single N-9 exposure but declines after repeated daily exposures

Previous studies of N-9 toxicity in the mouse revealed intense infiltration of CD45-positive immune cells and Ly6-positive neutrophils subsequent to a 2 h exposure to unformulated N-9 [19,25]. In preliminary experiments designed to expand these findings, mice were given a single application of unformulated 1% N-9 and sacrificed at 2, 4, or 24 h post-exposure. Cells isolated from excised cervicovaginal tissues before or after N-9 exposure were analyzed by flow cytometry to identify the following immune cell populations: CD4+/CD3+ T lymphocytes, CD8+/CD3+ T lymphocytes, Ly6G + neutrophils, and CD14+/CD11C- monocytes/macrophages. In addition to the abundance of infiltrating neutrophils following N-9 exposure, these experiments indicated a 50% increase in the number of CD14+/CD11c- cells at 2 h post-exposure (data not shown). In addition, monocyte/macrophage infiltration appeared to decrease by 4 h post-exposure and returned to control levels by 24 h post-exposure (data not shown).

**Figure 7:**
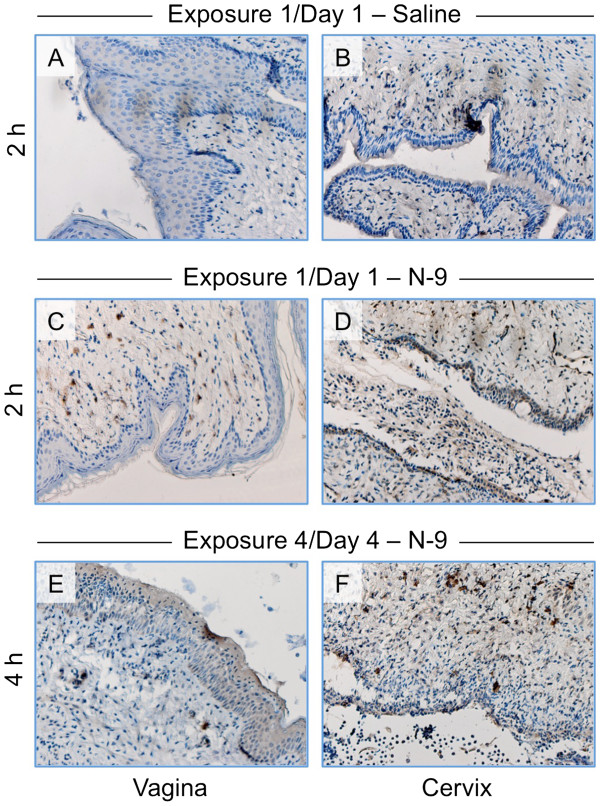
**N-9 exposure results in CD14**^**+**^**cell infiltration on day 1 and, to a lesser extent, on day 4.** Female Swiss Webster mice were exposed once to saline (**A**, **B**), once to unformulated 1% N-9 (**C**, **D**), or once daily to N-9 for four days (**E**, **F**). Mice were sacrificed after the indicated post-exposure intervals following the last exposure. Cervicovaginal tissue sections were stained immunohistochemically for the CD14 cell surface marker. Representative vaginal (**A**, **C**, **E**) and cervical (**B**, **D**, **F**) epithelial tissue sections are shown.

To localize macrophage/monocyte infiltration following single or multiple exposures to N-9 within the cervicovaginal epithelium and to confirm the preliminary findings, CD14+ cells were visualized immunohistochemically in exposed tissues in conjunction with a CD14-specific antibody. Analyses of control tissues exposed once to saline revealed little infiltration by CD14+ cell populations into the vaginal (Figure 7A) or cervical (Figure 7B) epithelium. In contrast, tissues excised at 2 h post-exposure following a single N-9 exposure (day 1) were characterized by light infiltration into the vaginal epithelium (Figure 7C) and intense CD14 staining under the epithelial surface in the cervix (Figure 7D). Levels of CD14+ cell infiltration in the cervix were less at 4 h post-exposure and declined considerably at 24 h post-exposure (data not shown). These results indicated that CD14+ cell infiltration was concurrent with peak N-9-associated physical damage within the cervical epithelium after a single exposure.

Because peak cervical epithelial damage after four daily N-9 exposures was observed at 4 h post-exposure (Figure 5 and Table 2), similar analyses were performed on cervicovaginal tissues collected at 4 h post-exposure from mice after four daily N-9 applications. As was observed after a single N-9 exposure, few CD14+ cells were observed in the day 4 vaginal epithelium (Figure 7E). In the cervical epithelium (Figure 7F), CD14+ cells were present in greater numbers relative to cell numbers in the corresponding vaginal tissues. However, cell infiltration was clearly not as intense as was noted at 2 h on day 1. Furthermore, the intense cervical sub-surface staining seen on day 1 was not apparent on day 4; infiltrating CD14+ cells were only present deeper in the lamina propria. CD14+ cell staining in the cervix at 4 h and 24 h post-exposure was also reduced relative to levels noted in cervical tissues on day 1 (data not shown). These results again indicate a parallel between physical epithelial damage and CD14+ cell infiltration within the cervix; apparent tolerance to N-9 exposure was accompanied by considerably lower levels of monocyte/macrophage infiltration.

## Discussion

Early efforts to develop a safe and effective microbicide ended with the observation that N-9, which had been used safely as a spermicidal agent for over four decades [9], was not only clinically ineffective against HIV-1 transmission, but was also capable of significantly increasing the risk of HIV-1 acquisition after repeated use [5]. These clinical trial results prompted a reassessment of mechanisms of N-9 toxicity and a general realization that a greater emphasis on pre-clinical microbicide safety was necessary.

One conclusion from post-failure analyses of N-9 microbicide development was that toxicity studies using the standard rabbit vaginal irritation (RVI) model provided an incomplete picture of the adverse effects of N-9 on the cervicovaginal epithelial tissues and the relevance of those effects to the risk of HIV-1 transmission. While the RVI model has provided important information regarding the safety of N-9 and other topical vaginal microbicides, there are limitations to the standard protocol. First, despite its demonstrated sensitivity to toxic topical agents, the RVI model does not assess product safety during the window of likely HIV-1 transmission. The standard RVI test protocol includes only one assessment of toxicity at 24 h after the final product application and does not provide for measures of toxicity at more acute post-exposure intervals, particularly the first hours after topical application when sexual intercourse and HIV-1 transmission are likely to occur. Our previous studies using the mouse model of cervicovaginal toxicity demonstrated that N-9- and C31G-mediated damage was greatest at 2 to 4 h post-exposure and was minimal or undetectable by 24 h post-application [19,24,25], presumably because epithelial repair mechanisms had restored the epithelium to its pre-exposure state. The standard RVI model would not reveal these important milestones in the time course of N-9 topical toxicity. Second, this model also differs from the human female reproductive tract (FRT) in that the rabbit FRT (i) does not undergo cyclic reproductive stages, (ii) is not colonized by lactobacillus (resulting in a lack of acidity within the vaginal tract), (iii) lacks the production of cervicovaginal mucus, and (iv) is characterized by a columnar epithelium in the upper vagina (cervicovagina) and a stratified squamous epithelium in the lower vagina (urovagina) [29-32].

We developed a Swiss Webster murine model [19] to assess cervicovaginal tissue integrity and inflammation following exposure to candidate vaginal microbicides and to specifically address the need for an *in vivo* model system that can be used to provide pre-clinical results predictive of clinical trial outcomes. The Swiss Webster mouse is a readily accessible, outbred stock strain that has previously been used as a model for studies of various infectious cervicovaginal tract pathogens, including *Chlamydia trachomatis*, herpes simplex virus, and group B streptococci (GBS). These mice have also been used in various pre-clinical microbicide studies [33-37]. The Swiss Webster mouse model offers several distinct advantages over the RVI model and other approaches used for the evaluation of cervicovaginal toxicity and inflammation associated with exposure to topical microbicides. First, the Swiss Webster mouse is a relatively inexpensive animal model, permitting large, pre-clinical toxicity screens of candidate compounds under a variety of experimental conditions (including multiple exposure protocols) that can be used to evaluate cervicovaginal toxicity and inflammation at the cellular and tissue level prior to Phase I safety trials. Second, previously published observations from experiments involving this model [19,25] have indicated close parallels to clinical findings, demonstrating the value of this model as a prescreening tool to prevent costly and time-consuming clinical trials on compounds with unacceptable safety profiles. Third, the mouse model, unlike the RVI model system, can be used to assess regional differences in cervicovaginal toxicity. This is an important feature of this animal model system, since topical toxicity can be tissue-specific, as we have demonstrated in past studies [19,25,38]. Consideration of regional differences in FRT toxicity may be relevant to understanding mechanisms of HIV-1 transmission, since non-human primate studies of SIV cervicovaginal infection suggest that HIV-1 transmission within the FRT may be regionally constrained [39,40], perhaps by the nature of the epithelial barrier or by regional differences in the distribution of HIV-1-susceptible immune cell populations [41]. The model, however, is not without its limitations: the lack of colonizing lactobacillus (addressed only in the non-human primate model); a higher cervicovaginal pH relative to the human FRT; and the use of Depo-Provera to pretreat the animals prior to experimentation.

The present studies, through the single N-9 exposure aspect of these experiments, have confirmed observations reported in previously published studies [19,25]. First, the cervical epithelium is severely damaged by a single exposure to N-9, while the vaginal epithelium remains relatively intact. The damage to the cervical epithelium is characterized by breaks in the columnar tissue architecture and severe tissue sloughing. Second, N-9-associated cervical epithelial damage occurs relatively rapidly. After a single exposure to 1% N-9, the damage to the cervical epithelium was greatest at 2 to 4 h post-exposure. Third, physical damage can be accompanied by intense immune cell infiltration. Unlike the present studies, past experiments identified the infiltrating cells as positive for CD45, which is a pan-leukocyte cell surface marker. Finally, the damage caused by a single N-9 exposure is transient and resolved over a period of approximately 24 h post-exposure.

The single exposure results also suggest that tissue damage may not be strictly associated with the anatomy of the cervicovaginal tract. We consistently observed severe N-9-associated damage to the columnar epithelium of the cervix and minimal damage to the stratified squamous epithelium of the lower vagina. However, one of the vaginal sections (Figure 3C, Day 2/Exposure 2, 2 h) shows intact stratified squamous epithelium adjacent to damaged columnar epithelium (roughly the lower half of the field), which is presumed to be part of the upper vaginal tract. Under Depo-Provera pre-treatment, the upper vaginal tract assumes a morphology typified by a single layer of columnar cells overlying 1–2 layers of basal cells. These observations indicate that epithelial damage subsequent to N-9 application is dependent on the architecture of the epithelial tissue rather than its anatomical location, and that a columnar epithelium, regardless of its location, is more susceptible to N-9 toxicity than a stratified squamous epithelium.

The present studies also provide new information regarding N-9 toxicity that is relevant to increases in HIV-1 transmission after repeated exposure to N-9. During repeated daily exposures to unformulated 1% N-9, the cervical epithelium appeared to become less sensitive to the degradative effects of N-9 application. By the fourth application, the damage caused by N-9 application was less and the peak damage was observed at 4 h post-exposure rather than at 2 h post-exposure as seen following the first application of N-9. This observation suggests the possibility that changes in the epithelial architecture after the initial exposure, insult, inflammation, and repair provide a protective mechanism against the effects of subsequent N-9 exposure.

This apparent tolerance to N-9 exposure after multiple applications may likely be related to changes in cervical tissue morphology observed during these studies. After multiple N-9 exposures, the tissue appeared to be metaplastic, forming multiple layers of stratified squamous epithelium instead of the usual single layer of columnar epithelium overlying one or two layers of basal epithelium. This effect has been observed subsequent to stress applied to tissue over time [42]. The increased tolerance of this metaplastic tissue is consistent with the above conclusion that susceptibility to N-9-associated damage is dependent on tissue architecture rather than anatomical location, since the stratified cervical tissue observed in day 4 mice was more tolerant of N-9 exposure compared to the columnar cervical epithelial tissue found in day 1 mice. One focus of future studies will be to determine the amount of time required for repaired and tolerant tissues to return to their baseline structures and levels of susceptibility to damage.

The present studies also revealed new information about relationships between repeated N-9-associated damage and induced tissue inflammation. Following N-9 exposure, the release of the pro-inflammatory cytokines IL-1β and IL-6 was detected in vaginal lavages, reinforcing the roles for these cytokines as important mediators of the inflammatory response in the vaginal tract in response to microbicide application [27,28]. However, only IL-6 release coincided with cervical epithelial damage (2–4 h post-exposure) after the initial N-9 application. In contrast, minimal amounts of IL-1β were detected on day 1. Conversely, IL-1β was released quickly (10 min post-exposure) and in relatively large amounts on day 4 relative to day 1. While IL-6 release was also detected on day 4, the magnitude of release was considerably less. In all cases, cytokine increases were transient and returned to control levels by 8 to 24 h post-exposure as tissue regeneration was in progress. Although the significance of these observations has not yet been determined, the pattern of cytokine release suggests an association with tissue regeneration following multiple N-9 exposures and the development of tolerance to N-9 application.

Subsequent to N-9 exposure, CD14+ monocytes/macrophages were also detected as part of the immune cell infiltrate. The intense, sub-surface presence of CD14+ immune cells after the first exposure to N-9 coincided with the cervical localization of epithelial damage, the peak severity of damage at 2 to 4 h post-exposure, and the detection of IL-6 in the vaginal lavage. The association between IL-6 release and the presence of CD14+ immune cells suggests that the source of the IL-6 may be the monocyte/macrophage population within the infiltrate [43,44]. This hypothesis is also consistent with the concomitant decrease in CD14+ cells and reduction in released IL-6 at 8 and 24 h post-exposure, and with the association between the reduced levels of IL-6 and lower numbers of infiltrating CD14+ cells on day 4.

## Conclusions

These studies provide new insights and raise new questions about cervicovaginal damage associated with multiple exposures to topical agents with epithelial toxicity. Future studies will need to explore several aspects of these results, including the underlying mechanisms of cervicovaginal epithelial regeneration and tolerance to toxic agents, and the involvement of the inflammatory response in the process of tissue recovery. Additional experiments will need to examine the effects of multiple exposures to toxic agents such as N-9 during a single day, since increases in the risk of HIV-1 acquisition were attributed to multiple uses of N-9 within a single 24 h period [5]. Finally and most importantly, these studies suggest the need for multiple exposure protocols in future safety and efficacy assessments during the pre-clinical development of topical vaginal (and rectal) microbicides effective against HIV-1 transmission.

## Competing interests

The authors declare that they have no competing interests.

## Authors’ contributions

KL established the study design, performed all experimental procedures, collected the data, analyzed the results, and prepared the manuscript. RO and BW participated in data analyses and the preparation of the manuscript. TKC contributed expertise in animal model studies and participated in data analyses. FCK participated in study planning, data analyses, and the preparation of the manuscript. All authors read and approved the final manuscript.

## Pre-publication history

The pre-publication history for this paper can be accessed here:

http://www.biomedcentral.com/2050-6511/13/9/prepub
